# Correlation of magnesium intake with metabolic parameters, depression and physical activity in elderly type 2 diabetes patients: a cross-sectional study

**DOI:** 10.1186/1475-2891-11-41

**Published:** 2012-06-13

**Authors:** Jui-Hua Huang, Yi-Fa Lu, Fu-Chou Cheng, John Ning-Yuean Lee, Leih-Ching Tsai

**Affiliations:** 1 Graduate Institute of Ph.D. Program in Nutrition and Food Science, Fu-Jen Catholic University, New Taipei City, Hsinchuang, Taiwan; 2 Department of Nutritional Science, Fu-Jen Catholic University, New Taipei City, Hsinchuang, Taiwan; 3 Department of Medical Research, Stem Cell Center, Taichung Veterans General Hospital, Taichung, Taiwan; 4 College of Living Technology, Tainan University of Technology, Tainan, Taiwan; 5 Division of Endocrine and Metabolism, Department of Internal Medicine, Erlin-Branch, Changhua Christian Hospital, Changhua, Taiwan; 6 Diabetes educational institute, Erlin branch, Changhua Christian Hospital, Changhua, Taiwan

**Keywords:** Magnesium, Diabetes, Metabolic control, Depression, Physical activity, Elderly

## Abstract

**Background:**

Type 2 diabetes mellitus is a major global public health problem in the worldwide and is increasing in aging populations. Magnesium intake may be one of the most important factors for diabetes prevention and management. Low magnesium intake may exacerbate metabolic abnormalities. In this study, the relationships of magnesium intake with metabolic parameters, depression and physical activity in elderly patients with type 2 diabetes were investigated.

**Methods:**

This cross-sectional study involved 210 type 2 diabetes patients aged 65 years and above. Participants were interviewed to obtain information on lifestyle and 24-hour dietary recall. Assessment of depression was based on DSM-IV criteria. Clinical variables measured included anthropometric measurements, blood pressure, and biochemical determinations of blood and urine samples. Linear regression was applied to determine the relationships of magnesium intake with nutritional variables and metabolic parameters.

**Results:**

Among all patients, 88.6% had magnesium intake which was less than the dietary reference intake, and 37.1% had hypomagnesaemia. Metabolic syndromes and depression were associated with lower magnesium intake (*p <* 0.05). A positive relationship was found between magnesium intake and HDL-cholesterol (*p* = 0.005). Magnesium intake was inversely correlated with triglyceride, waist circumference, body fat percent and body mass index (*p* < 0.005). After controlling confounding factor, HDL-cholesterol was significantly higher with increasing quartile of magnesium intake (*p* for trend = 0005). Waist circumference, body fat percentage, and body mass index were significantly lower with increase quartile of magnesium intake (*p* for trend < 0.001). The odds of depression, central obesity, high body fat percentage, and high body mass index were significantly lower with increasing quartile of magnesium intake (*p* for trend < 0.05). In addition, magnesium intake was related to high physical activity level and demonstrated lower serum magnesium levels. Serum magnesium was not significantly associated with metabolic parameters.

**Conclusions:**

The majority of elderly type 2 diabetes who have low magnesium intake may compound this deficiency with metabolic abnormalities and depression. Future studies should determine the effects of increased magnesium intake or magnesium supplementation on metabolic control and depression in elderly people with type 2 diabetes.

## Background

Type 2 diabetes mellitus (DM) is a major public health problem in the worldwide and is increasing in aging populations [1,2]. Moreover, type 2 DM is a significant cause of premature mortality and morbidity related to cardiovascular disease, macrovascular complications, and microvascular complications in older adults [3-5]. The optimal goal of management in DM is to achieve and maintain adequate blood glucose, blood lipids, and blood pressure levels to prevent or delay chronic complications of diabetes. Elderly patients with diabetes can achieve adequate metabolic control by following a nutrition plan and an exercise program, losing excess weight, complying with a self-management education intervention program and taking appropriate medications [6].

Magnesium may be one of the most important factors for diabetes prevention and management [7,8]. Indeed, magnesium has been associated with a number of chronic disease including, diabetes, hypertension, insulin resistance and lipid abnormalities [9]. Magnesium deficiency (1) is a common factor associated with insulin resistance and vascular disease [10], (2) impairs energy metabolism efficiency and reduces the capacity for physical work [11], (3) exerts negative effects on blood glucose homeostasis [12], and (4) is independently associated with depressive symptoms [13]. In addition, magnesium intake was inversely associated with the metabolic syndrome and risk of type 2 DM [8,14]. High plasma triglycerides, abdominal adiposity, albuminuria and low HDL-cholesterol are associated with hypomagnesemia in diabetic patients [15,16]. Double-blind placebo-controlled trials suggest magnesium supplementation may (1) improve insulin sensitivity, reduce of plasma cholesterol and LDL cholesterol, increase of HDL cholesterol in DM patients [7,17] (2) lower blood pressure in patients with essential hypertension [18], and (3) improve resting ionic magnesium levels in physically active women [19]. Furthermore, magnesium supplementation has been shown to be as effective as an antidepressant drug for treatment of depression in the elderly DM patients with hypomagnesemia [20].

Assessment of magnesium status is important for determining the association of magnesium with metabolic control and cardiovascular disease. A simple method available in a clinical setting is the measurement of serum magnesium. However, serum magnesium levels correlate poorly with magnesium status [21]. The determination of intracellular magnesium levels from muscle biopsy, lymphocytes, and red blood cells is a much better predictor of magnesium status via nuclear magnetic resonance spectroscopy or ion-specific electrode measures [22]. These tests are of value in basic research; however, they are expensive and impractical. Several studies showed that magnesium intake is associated with diabetes risk, metabolic control and cardiovascular disease [8,14,23]. In general, assessment of magnesium intake may be not accurate. However, a more effective assessment of dietary intake can be achieved through improved methodology and interview, and such evaluations offer the advantage of being non-invasive [24].

Numerous clinical studies on magnesium supplementation have suggested that dietary magnesium deficiency may impair metabolic control [10,25]. However, various studies of magnesium intakes and magnesium supplementation were inconsistent [7,17]. Indeed, dietary magnesium intake in elderly patients with diabetes and its relationship to metabolic control, depression and physical activity have not been intensively explored.

The present study was conducted to investigate magnesium status in elderly type 2 diabetes patients and to determine its relationship with nutritional variables, metabolic control, depression and physical activity. In this cross-sectional study, information about dietary intake, physical activity, and depression were obtained by questionnaires. In additional, anthropometric values, as well as biochemical determinations of blood and urine samples were also investigated.

## Subjects and methods

### Study subjects

The cross-sectional study was approved by the Changhua Christian Hospital Institutional Review Board (CCHIRB#090419). Patients were residents in a rural area of central Taiwan, and diabetes at the first the Endocrine and Metabolism clinic was diagnosed according to the American Diabetes Association (ADA) criteria [26]: 1). fasting plasma glucose (FPG) ≧126 mg/dL. Fasting is defined as no caloric intake for at least 8 h. OR 2). 2-h plasma glucose ≧200 mg/dL during an oral glucose tolerance test (OGTT). OR 3). in a patient with classic symptoms of hyperglycemia or hyperglycemic crisis, a random plasma glucose≧200 mg/dL. In general, patients with type 2 diabetes commonly have components of metabolic syndrome (e.g. dyslipidemia and hypertension). The medication use is following the Taiwan Diabetes Clinical Care Guideline, the ADA standards of medical care in diabetes [27] and the National Cholesterol Education Program (NCEP) Expert Panel on Detection, Evaluation, and Treatment of High Blood Cholesterol in Adults (Adult Treatment Panel III) [28,29]. The inclusion criteria for patients were as follows: *1*) Type 2 diabetes for more than 6 months; *2*) no change in any medications for the past 3 months; *3*) stable lifestyle for the past 3 months; and; *4*) aged 65 years and above. Patients with heart failure, cirrhosis, current malignancy, chronic renal failure, or signs of serious deterioration in comprehension and memory were excluded. Anthropometric measurements, biochemical blood and urine tests, as well as dietary assessment were performed within one month. For elderly patients with a low level of education and who were unable to write their signature, children or family members signed consent forms on their behalf.

### Dietary assessment

Dietary intake was assessed using 24-hour recall [24,30]. In addition, questionnaires of typical dietary pattern were also applied in the survey to assess subjects’ daily dietary intake. The 24-h dietary recalls and a week of typical dietary pattern were collected via interview by a registered dietitian. During the interview, quantitative tools including standard measuring spoons and cups, food models, food pictures and photos, and traditional household bowls, cups and spoons were used to help the these elderly subjects [31]. Magnesium and other nutrients intake were analyzed using Taiwan Nutrition Database and the EKitchen nutritional analysis software (Nutritional Chamberlain Line, Professional Edition, version 2001/ 2003, EKitchen Inc, Taichung, Taiwan) [32]. Taiwan Dietary Reference Intakes (DRI) for magnesium in adults and elderly person was based on the U.S.A. EAR of 5 mg/kg/day [33] and Taiwanese reference body weight. Therefore, Taiwan DRI of magnesium for health individuals above 19 years of age is 360 mg/d for males and 315 mg/d for females. Taiwan RDA for protein in elderly person is 1 g//kg/day [34]. In this study, we divided protein intake into 3 categories and defined the following ranges: < 0.8, 0.8 to 1.0, > 1.0 g//kg. In addition, the Taiwan Resting Energy Expenditure (REE) for energy in aged 65 years and above is 22 kcal/kg/day for men or 20 kcal/kg/day for women. We referred to Taiwan REE and estimated energy requirement divided energy intake into 3 categories and defined the following ranges: < 25, 25 to 30, >30 kcal//kg.

### Assessment of physical activity

Assessment of physical activity is based on a slightly modified version of the method using in a Finnish study [35]. Briefly, occupational physical activity was divided into: 1) light: physically very easy, seated office work; 2) moderate: work including standing and walking; and 3) active: work including walking and lifting, or heavy manual labor. Daily commuting journey was categorized into: 1) using motorized transportation, or no work; 2) walking or bicycling 1–29 min; and 3) walking or bicycling > 30 min. Self-reported leisure-time physical activity was classified into: 1) low: almost completely inactive; 2) moderate: some physical activity >4 h per week; and 3) high: vigorous physical activity > 3 h per week. Physical activities were accumulated and regrouped into: 1) low; 2) moderate and 3) high according to the Finnish study [35].

### Assessment of depression

Assessment of depression was based on the criteria in Diagnostic and Statistical Manual of Mental Disorders, Fourth Edition (DSM-IV), and included the number and severity of symptoms, duration of the current episode, and course of illness. Depression was diagnosed in patients with 5 or more symptoms including at least one key symptom, most days, most of the time for at least 2 weeks, as defined by the American Psychiatric Association [36].

### Biochemical determinations of blood and urine

For biochemical determinations of blood, **s**ubjects were asked to fast for 8–10 hours on the night prior to measurement. Study measurements were performed by the hospital medical laboratory (certified ISO15189) using standard methods. The glycated hemoglobin (HbA1C) (CV < 3.0%) was measured by ion-exchange chromatography (BioRad Variant, Hercules, California, USA). Serum magnesium, albumin, lipids, urea nitrogen, creatinine, high-sensitivity CRP (hsCRP), microalbumin using a Dimension RxL autoanalyzer (Siemens, Newark, USA). Serum magnesium (CV < 3.0%) was measured using the methylthymol blue method. Serum albumin (CV < 2.0%) was estimated using the bromcresol purple method. Lipids [total cholesterol (CV < 3.0%), HDL (CV < 4.0%), LDL (CV < 4.0%), triglyceride (CV < 3.0%)] were assessed using standard enzymatic methods. The high-sensitivity C-reactive protein (hsCRP) (CV < 3.0%) was measured by particle enhanced turbidimetric immunoassay. The levels of creatinine (CV < 2%) in urine and serum were used the alkaline picrate-kinetic method. Blood urea nitrogen (CV < 3.0%) was measured using the methylthymol blue method. Estimated glomerular filtration rate (eGFR) was calculated as eGFR (mL/min/1.73 m^2^) (Simplified Modification of Diet in Renal Disease (MDRD)) = 186× serum creatinine^-1.154^ × Age^-0.203^ in men, and 186× serum creatinine^-1.154^× Age^-0.203^× 0.742 in women according to the formula recommended by the Taiwan Society of Nephrology.

### Anthropometric measurements

Anthropometric measurements included height, weight, blood pressure, waist circumference (WC) and body fat percentage. As for blood pressure measurement, prior to measurement, the subject removed any clothing in the arm area and was left to rest for five minutes to avoid stimulation. A mercury sphygmomanometer was used, which was held at the same height as the arm and heart during measurement, with palms facing upward. Percentage of body fat was estimated with bioelectrical impedance analysis using TBF-410 (TANITA, Tokyo, Japan). Body mass index (BMI) was also calculated in weight (kg)/height (m^2^).

### Diagnostic criteria for blood, urine, blood pressure and anthropometric measurements

Clinical criteria for blood, urine and anthropometric measurements were defined the following ranges: 1) patients with hypomagnesaemia, defined as serum magnesium < 0.75 mmol/L [22]; 2) goals of glycemic, lipid and blood pressure control recommend as HbA1C < 7.0%, HDL-cholesterol > 50 mg/dL in females and > 40 mg/dL in males, triglyceride <150 mg/dL and blood pressure < 130/80 mmHg [28]; 3) WC has set at ≧ 80 cm for men or ≧ 90 cm for women were categorized as abdominal adiposity. Body fat percentages >30% for females and >25% for males were categorized as obese. BMI value for elderly people exceeding 27 results in dramatically elevated mortality risk of diabetes [37]. 4) the diagnosis of metabolic syndrome was made following either the National Cholesterol Education Program-Adult Treatment Panel (NCEP-ATPIII) or the International Diabetes Federation (IDF) criteria. According to both sets of criteria, metabolic syndrome is present when at least three of five conditions (abdominal adiposity, hyperglycemia, reduced HDL- cholesterol, elevated triglyceride, and increased arterial blood pressure) are satisfied [38]; 5) hsCRP levels of <1, 1 to 3, and >3 mg/L be used to represent lower, moderate, and higher vascular risk [39]; and 6) stages of chronic kidney disease (CKD) were defined as follows: stage 1 (eGFR ≥ 90 mL/min/1.73 m^2^); stage 2 (eGFR 60–89 mL/min/1.73 m^2^); stage 3 (eGFR 30–59 mL/min/1.73 m^2^); stage 4 (eGFR 15–29 mL/min/1.73 m^2^); and stage 5 (eGFR <15 mL/min/1.73 m^2^) [40].

### Statistical analysis

Data were analyzed by 2-tailed t-test and one-way ANOVA for continuous variables and frequency distribution for categorical variables. Correlation of magnesium intake levels with metabolic parameter, body fat percentage and body mass index were examined in multiple linear regression analysis. Tests for linear trend were conducted by using the statement contrast in linear regression models. Logistic regression models were used to examine the relationship between magnesium intake levels with depression, metabolic risk factors, high body fat percentage and high body mass index status. Data are expressed as odds ratios and 95% CIs. Tests for linear trend were conducted by modeling the odds of each quartile -defined category of magnesium intake as a continuous variable in logistic regression models. All statistical procedures were performed using SPSS (version 17.0) statistical software, (SPSS Inc., Chicago, IL, USA), and a p-value of 0.05 was considered statistically significant.

## Results

The clinical characteristics of the elderly patients with type 2 DM are shown in Table 1. Of the 210 patients, 88.6% had magnesium intake less than the dietary reference intake (DRI), and 37.1% had hypomagnesemia (serum magnesium < 0.75 mmol/L). Magnesium intake and serum magnesium by dietary intake, physical activity level, depression, metabolic parameters, body fat percentage, BMI, hsCRP and eGFR status are shown in Table 2. Lower intake of energy and protein were significantly associated with lower magnesium intake (*p* < 0.001). The low physical activity group displayed lower magnesium intake than those of the moderate and high physical activity group (*p* = 0.002). However, the high physical activity group displayed lower serum magnesium levels than those of the moderate physical activity group (*p* = 0.024). Compared with non-depressed patients, depression patients consumed significantly lower magnesium intake (*p* = 0.001). Mean magnesium intakes were significantly lower in patients with low HDL (*p <* 0.001), central obesity (*p* = 0.051), high body fat percent (*p* = 0.000) and high BMI (*p <* 0.001). Compared with non- metabolic syndromes patients, patients with metabolic syndromes displayed significantly lower magnesium intake (*p* = 0.013). Estimated GFR showed marginal positivity relationships with magnesium intake (*p* = 0.067). Magnesium intake was not significantly correlated with serum albumin, HbA1C, blood pressure, triglyceride or hsCRP. However, serum magnesium levels were not significantly associated with other variables.

**Table 1:** Characteristics of 210 elderly patients with type 2 diabetes

**Variables**	**Mean ± SD**	**n (%)**
Age (y)	72.3 ± 5.4	
Gender		
Men		98 (46.7)
Female		112 (53.3)
Diabetes duration (y)	10.9 ± 7.6	
Diabetes medication		
Oral hypoglycemic drug		147 (70.0)
Insulin and Oral hypoglycemic drug		63 (30.0)
Dietary intake		
Energy intake (kcal/kg)	26.0 ± 7.8	
Protein intake (g/kg)	0.8 ± 0.3	
Magnesium intake (mg/kg)	3.6 ± 1.7	
Magnesium intake < DRI		186 (88.6)
Serum magnesium (mmol//L)	0.78 ± 0.09	
Hypomagnesaemia ( < 0.75 mmol//L)		78 (37.1)
Serum albumin (g/dL)	4.0 ± 0.3	

1. Taiwan DRI (dietary reference intakes, DRI) of magnesium for health individuals above 19 years of age is 360 mg/d for males and 315 mg/d for females.

2. Categorical variables were analyzed according to frequency distribution and expressed in number and percentages [n (%)].

3. Continuous variables were presented as mean values and standard deviation (Mean ± SD).

**Table 2:** Magnesium intake and serum magnesium by dietary intake, physical activity level, depression, metabolic parameter, body fat percentage, body mass index, hsCRP and estimated GFR status

**Variables**	**n**	**Mg intake (mg/kg)**	***P***	**Serum Mg (mmol/L)**	***P***
Dietary intake					
Energy intake (kcal/kg)			0.000		0.636
< 25	109	2.8 ± 1.3 ^ab^		0.77 ± 0.10	
25 to 30	47	4.0 ± 1.2 ^a^		0.79 ± 0.10	
>30	54	4.6 ± 2.0 ^b^		0.79 ± 0.08	
Protein intake (g/kg)			0.000		0.172
< 0.8	115	2.9 ± 1.3 ^ab^		0.77 ± 0.10	
0.8 to 1.0	48	3.7 ± 1.2 ^ac^		0.80 ± 0.09	
>1.0	47	5.0 ± 2.0 ^bc^		0.79 ± 0.08	
Physical activity level			0.002		0.024
Low	48	2.8 ± 1.4 ^a b^		0.79 ± 0.11	
Moderate	83	3.8 ± 1.7 ^a^		0.80 ± 0.09 ^c^	
High	79	3.8 ± 1.7 ^b^		0.76 ± 0.08 ^c^	
Depression (DSM-IV)			0.001		0.554
< 5 symptoms	147	3.8 ± 1.7		0.78 ± 0.10	
≧ 5 symptoms	63	3.0 ± 1.4		0.78 ± 0.09	
Metabolic parameters					
HbA1c (%)			0.709		0.558
< 7.0	103	3.6 ± 1.6		0.79 ± 0.08	
≧ 7.0	107	3.5 ± 1.7		0.78 ± 0.11	
HDL-cholesterol (mg/dL)			0.051		0.659
≧ 40 for men or ≧ 50 for women	114	3.8 ± 1.8		0.78 ± 0.09	
< 40 for men or < 50 for women	96	3.3 ± 1.5		0.78 ± 0.10	
Triglycerides (mg/dL)			0.452		0.456
< 150	172	3.6 ± 1.7		0.78 ± 0.10	
≧ 150	38	3.4 ± 1.4		0.79 ± 0.08	
Blood pressure (mmHg)			0.344		0.669
Systolic BP < 130 / Diastolic BP < 80	64	3.7 ± 2.0		0.79 ± 0.09	
Systolic BP ≧ 130 / Diastolic BP ≧ 80	146	3.5 ± 1.5		0.78 ± 0.10	
Waist circumference (cm)			0.000		0.687
< 80 for men or < 90 for women	44	4.6 ± 2.2		0.78 ± 0.10	
≧ 80 for men or ≧ 90 for women	166	3.3 ± 1.4		0.78 ± 0.09	
Metabolic syndrome			0.013		0.234
< 3 components	54	4.2 ± 2.3		0.80 ± 0.10	
≧ 3 components	156	3.3 ± 1.4		0.78 ± 0.09	
Body Fat percentage (%)			0.000		0.873
< 25 for men or < 30 for women	101	4.0 ± 1.9		0.78 ± 0.09	
≧ 25 for men or ≧ 30 for women (obese)	109	3.1 ± 1.4		0.78 ± 0.10	
Body mass index (kg/m^2^)			0.000		0.803
< 27	151	3.9 ± 1.7		0.78 ± 0.10	
≧ 27 (obese)	59	2.8 ± 1.3		0.78 ± 0.09	
High-sensitivity CRP (mg/L)			0.246		0.186
< 1 Low risk	57	3.9 ± 1.9		0.76 ± 0.10	
1 to 3 Moderate risk	105	3.5 ± 1.6		0.79 ± 0.10	
> 3 High risk	35	3.3 ± 1.5		0.80 ± 0.08	
Estimated GFR(ml/min)			0.067		0.363
≧ 90 CKD stage 1	30	4.0 ± 1.7		0.76 ± 0.10	
60 - 89 CKD stage 2	121	3.6 ± 1.8		0.79 ± 0.09	
30 - 59 CKD stage 3	59	3.1 ± 1.4		0.78 ± 0.11	

1. Abbreviations: HbA1C, glycated hemoglobin; HDL, high-density lipoprotein; BP, blood pressure; CRP, C-reactive protein; GFR, glomerular filtration rate; CKD, chronic kidney disease.

2. The *t* test was used for the difference in the means of two groups. The means of more than two groups were compared using one-way ANOVA followed by Scheffe’s multiple comparisons test. ^a-a^, ^b-b^, ^c-c^ indicates significant difference between two groups. Data are means ± SD. Significant difference (*p* < 0.05).

Simple linear regression was applied to determine the relationship of magnesium intake to serum magnesium, nutritional variables, metabolic parameters, body fat percent, body mass index, hsCRP, and eGFR. Serum magnesium levels were not significantly correlated with magnesium intake. Magnesium intake had positive association with energy intake (r 0.520; *p* = 0.000) and protein intake (r 0.646; *p* = 0.000). A borderline positive relationship was observed between magnesium intake and serum albumin (r 0.117; *p* = 0.092). Furthermore, magnesium intake was positively correlated with HDL (r 0.192; *p* = 0.005). Magnesium intake had significant inverse relationships with triglyceride (r - 0.144; *p* = 0.037) and WC (r - 0.243; *p* = 0.000). Magnesium intake showed marginal inverse associations systolic blood pressure (r - 0.123; *p* = 0.074) and diastolic blood pressure (r - 0.128; *p* = 0.065). However, there was no significant relationship between magnesium intake and HbA1C. In addition, magnesium intake had an inverse relationship with body fat percentage (r - 0.149; *p* = 0.031) and BMI (r – 0.289; *p* = 0.000). A marginal positive relationship were observed between magnesium intake and eGFR (r 0.126; *p* = 0.068). No significant relationship was found between magnesium intake and hsCRP.

These data were also analyzed and stratified by sex (data not shown). The information remains about the same. For both men and female, lower magnesium intake was significantly associated with lower intake of energy and protein and high body mass index (*p* < 0.05). High physical activity demonstrated lower serum magnesium levels in men and female (*p* < 0.05). In addition, man and female were similar to unstratified analysis that serum magnesium was not significantly correlated with energy intake, protein intake, and eGFR, metabolic parameter, body fat percentage and body mass index. For the elderly men with Type 2 diabetes, magnesium intake was significantly lower in patient with metabolic syndrome (0.022). Physical activity level was significantly associated with magnesium intake in men (*p* = 0.010). However, physical activity level were not significantly associated with magnesium intake in female (*p* = 0.218). For the elderly female with Type 2 diabetes, magnesium intake was significantly lower in patient with depression (*p* = 0.003). Central obesity showed marginal inverse relationships with magnesium intake (*p* = 0.056).

Adjusted mean of metabolic parameters, body fat percentage and body mass index and adjusted Odd ratio of depression and metabolic risk factors by quartile of magnesium intake after controlling confounding factors are shown in Table 3. Adjusted mean of HDL was significantly higher with increasing quartile of magnesium intake (*p* for trend *=* 0.006). In addition, adjusted mean of waist circumference, body fat percentage, and body mass index were significantly lower with increasing quartile of magnesium intake (*p* for trend *<* 0.001). The adjusted mean of systolic blood pressure was marginal linear trend with quartile of magnesium intake (*p* for trend *<* 0.089). The adjusted mean of triglyceride and diastolic blood pressure was not significantly linear trend with quartile of magnesium intake, but the adjusted mean of triglyceride and diastolic blood pressure were lower with increasing quartile of magnesium intake (*p* for trend *<* 0.120). The odds of depression, central obesity, high body fat percentage, and high body mass index were significantly lower with increasing quartile of magnesium intake (*p* for trend *<*0.005). Although the odds of low HDL and metabolic syndrome was not significantly linear trend with quartile of magnesium intake, the odds of low HDL and metabolic syndrome were lower with increasing quartile of magnesium intake (*p* for trend *<* 0.165). In addition, adjusted mean of metabolic parameters, body fat percentage and body mass index and adjusted Odd ratio of depression and metabolic risk factors was not significantly correlated with levels of serum magnesium.

**Table 3:** Adjusted mean of metabolic parameters, body fat percentage and body mass index and adjusted Odd ratio of depression and metabolic risk factors by quartile of Mg intake

**Variables**	**Quartile of Mg intake**				***P* for**
	**Q1 (n = 52)**	**Q2 (n =53 )**	**Q3 (n =52 )**	**Q4 (n =53 )**	**trend**
Quartile range of Mg intake (mg/kg)	< 2.3	2.3 - 3.2	3.3 - 4.4	≧4.5	
Mean Mg intake (mg/kg) ^2^	1.8 ± 0.4	2.8 ± 0.3	3.7 ± 0.3	5.8 ± 1.4	<0.001
Adjusted mean ^3^					
Metabolic parameters^*^					
HbA1c (%)	7.3 ± 1.5	7.5 ± 1.5	7.2 ± 0.9	7.2 ± 1.2	0.838
HDL-cholesterol (mg/dL)	45.3 ± 11.7	44.4 ± 10.2	47.5 ± 13.3	50.6 ± 12.9	0.006
Triglycerides (mg/dL)	113.0 ± 51.3	113.5 ± 77.5	106.4 ± 15.4	92.0 ± 48.8	0.104
Systolic BP	137.1 ± 15.3	136.2 ± 14.7	135.4 ± 15.4	132.9 ± 13.7	0.089
Diastolic BP	78.6 ± 14.4	77.6 ± 11.7	77.8 ± 14.4	74.9 ± 12.5	0.117
Waist circumference (cm)	95.6 ± 9.4	92.9 ± 8.0	91.0 ± 9.9	88.8 ± 8.1^c^	<0.001
Body Fat percentage (%)^*^	33.0 ± 10.8	29.4 ± 9.0	28.8 ± 6.8	24.5 ± 8.5	<0.001
Body mass index (kg/m^2^) ^*^	27.2 ± 4.3	25.3 ± 3.4	25.1 ± 3.3	23.6 ± 3.0	<0.001
Adjusted Odds ratio ^4^					
Depression (DSM-IV)≧5 symptoms ^†^	1.00	0.37 (0.16-0.88)	0.44 (0.19-1.02)	0.22 (0.08-0.57)	0.003
Metabolic parameters^*^					
HbA1c (%)≧7.0	1.00	1.01 (0.41-2.50)	1.33 (0.53-3.33)	0.95 (0.34-2.66)	0.907
HDL-cholesterol (mg/dL) < 40 for men or < 50 for women	1.00	1.33 (0.57-3.10)	0.99 (0.41-2.36)	0.54 (0.20-1.44)	0.162
Triglycerides (mg/dL)≧150	1.00	1.51 (0.49-4.65)	1.91 (0.61-5.99)	0.90 (0.23-3.48)	0.963
Systolic BP≧130 /Diastolic BP≧80	1.00	0.84 (0.34-2.09)	1.04 (0.40-2.73)	0.60 (0.21-1.70)	0.452
Waist circumference (cm) ≧80 for men or ≧90 for women	1.00	0.41 (0.11-1.54)	0.57 (0.14-2.27)	0.09 (0.02-0.37)	0.001
Metabolic syndrome	1.00	2.10 (0.73-6.06)	1.68 (0.60-4.70)	0.49 (0.17-1.43)	0.153
Body fat percentage (%)≧25 for men or ≧30 for women (obese) ^*^	1.00	0.80 (0.32-2.05)	0.55 (0.21-1.44)	0.21 (0.07-0.61)	0.004
Body mass index (kg/m^2^)≧27 (obese) ^*^	1.00	0.63 (0.26-1.53)	0.21 (0.07-0.61)	0.09 (0.02-0.35)	<0.001

1. Abbreviations: Mg, magnesium; HbA1C, glycated hemoglobin; HDL, high-density lipoprotein; BP, blood pressure.

2. The means of four groups were compared using one-way ANOVA. Data are means ± SD. Significant difference (*p* < 0.05).

3. Correlation of Mg intake levels with metabolic parameters, body fat percentage and body mass index were examined in multiple linear regression analysis. Data are adjusted mean ± standard error (SE). Tests for linear trend were conducted by using the statement contrast in linear regression models. Statistically significant at *p* <0.05.

4. Logistic regression models were used to examine the relationship between Mg intake levels with depression, metabolic risk factor, high body fat percentage and high body mass index status. Data are odds ratios (95% CIs). Tests for linear trend were conducted by modeling the odds of each quartile-defined category of magnesium intake as a continuous variable in logistic regression models. Statistically significant at *p* <0.05.

5. confounding factors.

^*^ indicates adjusted for sex, age, physical activity level, total energy intake (kcal/day), carbohydrate intake (% of energy), protein intake (% of energy), total fat intake (% of energy), smoking, and alcohol consumption.

^†^ indicates adjusted for sex and age.

## Discussion

Our data suggest that (1) the majority of elderly type 2 diabetes patients may have low magnesium intake; (2) about one-third of elderly diabetes patients had hypomagnesaemia; (3) low magnesium intake was associated with metabolic abnormalities and depression; (4) magnesium intake was related to high physical activity level and demonstrated lower serum magnesium levels. However, serum magnesium was not significantly associated with metabolic parameters.

In general, dietary magnesium does not meet the DRI for elderly subjects and may be prone to chronic latent magnesium deficiency [41,42]. Daily magnesium intake for males and females above 50 years of age was 326 mg and 255 mg, respectively, in the United States. Furthermore, 60-62% of middle-aged to elderly people do not meet the DRI for magnesium [42]. The average magnesium intake for male and female elders was 279 mg and 227 mg, respectively, according to data from the Nutrition and Health Surveys in Taiwan [41]. These data indicated that they are at least 87% of the population in Taiwan do not meet the DRI for magnesium. In our data, daily consumption of magnesium in male and female elderly type 2 DM patients was only 253 mg and 189 mg magnesium, respectively. This indicates that 89% of our patients do not meet the DRI for magnesium.

It is generally accepted that magnesium deficiency is closely related to metabolic syndromes. Increased dietary magnesium intake and/or magnesium supplementation may improve metabolic syndromes and type 2 DM [14,25]. Our data also revealed that lower magnesium intake was correlated with metabolic syndromes. It affects HDL, triglyceride, WC, BMI, and body fat percentage. In addition, systolic and diastolic blood pressure showed marginal inverse relationships with magnesium intake. The overall prevalence of metabolic syndromes in these rural-dwelling elderly type 2 DM patients was 74% which was much higher than rates reported in two previous studies on healthy older adults (40%) [14,43]. However, the relationships of magnesium intake with blood glucose and lipids were inconsistent. Thus, increased magnesium intake may improve metabolic control in patients with metabolic syndromes.

Depression is the fourth-leading cause of disability worldwide according to the World Health Organization [44]. Prevalence rates of depression are between 5% and 10% [45], whereas it is estimated to be about 12% to 18% in patients with diabetes [46]. Moreover, 31% of elderly diabetes was found to have severe depressive symptoms [47]. This finding was confirmed and 30% of elderly diabetes patients had depression, particularly in those with low magnesium intake. Indeed, magnesium deficiency has been independently associated with depressive symptoms [13]. A recent randomized, equivalent trial suggests that oral magnesium supplementation is as effective as imipramine in the treatment of depressed elderly diabetes with hypomagnesemia [20]. Thus, increasing dietary magnesium intake or magnesium supplementation for elderly patients with diabetes may benefit the treatment of depression.

A review study indicated that 14-48% of diabetes suffered from hypomagnesaemia [48]. In the present study, the prevalence of hypomagnesaemia was about 37%. In addition, we found that magnesium intake had a significant positive relationship with energy intake and protein intake. Moreover, lower intake of energy and magnesium was correlated with a higher prevalence of hypomagnesemia. In the present study, prevalence of hypomagnesemia for the energy intake < 25 kcal/kg, 25–30 kcal/kg, and > 30 kcal/kg groups were 40%, 36%, and 32%, respectively. Likewise, lower intake of protein and magnesium was associated with a higher prevalence of hypomagnesemia. Prevalence of hypomagnesemia for the protein intake < 0.8 g/kg, 0.8-1.0 g/kg and > 1.0 g/kg groups were 43%, 27%, and 34%, respectively. However, 89% of the elderly diabetes patients had a magnesium intake which was less than the DRI, however, the prevalence of hypomagnesaemia was only 37%. Therefore, more than 50% of our patients with a magnesium intake less than the Taiwan DRI could not be identified as hypomagnesemia (low serum magnesium <0.75 mmol/L). Therefore, our results revealed that serum magnesium was not correlated well with metabolic parameters and depression as shown in a previous study [49]. Many studies showed that low magnesium intake is associated with certain degrees of diabetes risk, metabolic control and cardiovascular diseases. Serum magnesium may not be a good marker and may be inaccurate to correlates with magnesium status. However, hypomagnesemia could indicate a higher possibility of critical situations associated to diabetes, metabolic syndromes or cardiovascular diseases. Future clinical trials need to reveal relationships of serum magnesium, intracellular magnesium levels with various diseases.

The relationship between magnesium status and physical activity has received much attention in the past decade [11]. Our data clearly indicated that the low physical activity group had lower magnesium intake. Magnesium intake was about the same in the high and moderate physical activity groups. However, the high physical activity group had a significantly lower serum magnesium levels. In our data, prevalence of hypomagnesemia in the low, moderate and high physical activity groups were 27%, 31% and 49% respectively. Obviously, the high physical activity groups also had a significantly higher prevalence of hypomagnesemia when compared with that in other two physical activity groups. Thus, high intensity physical activity with inadequate magnesium intake seems to increase the risk of hypomagnesemia. This may be partly due to increased magnesium loss in perspiration (especially when working or exercising in hot environments) and in urine [50]. This could result in increased magnesium excretion and might increase the need for higher magnesium intake. When magnesium intake is insufficient, physical activity will exacerbate the magnesium deficiency [51]. Increased dietary magnesium intake or magnesium supplementation has beneficial effects on physical performance in magnesium-deficient individuals [52,53]. Hence, for elderly diabetes patients with high physical activity and low serum magnesium, it is necessary to increase dietary magnesium intake or magnesium supplementation.

There were several limitations of this study. First, the analyses were highly dependent on self-reported dietary intake and lifestyle data. Overestimation, underestimation, and poor recall might therefore have confounded the results. Fortunately, these elderly DM patients lived in rural areas and mostly of them had a simple lifestyle and eating patterns. The use of traditional quantitative tools, food models, food pictures and photos helped these elderly patients to recall the amount of consumed food and may also increase the effectiveness of the 24-hour recall. Moreover, questionnaires were also used to valid dietary patterns during the previous week. Second, serum magnesium may not be sufficient for indicating magnesium deficiency [21]. Therefore, the majority of patients (>89%) with less than the DRI magnesium intake were not identified as magnesium deficiency from their serum magnesium levels. Further investigation will be warranted.

## Conclusions

Majority of elderly diabetes patients have low magnesium intake and may exacerbate metabolic abnormalities and depression. Clinical care should therefore focus on increasing dietary magnesium intake or magnesium supplementation to improve metabolic control, depression, and physical performance in elderly diabetes patients.

## Abbreviations

BMI, Body mass index; BP, Blood pressure; CKD, Chronic kidney disease; DRI, Dietary reference intakes; DSM-IV, Diagnostic and Statistical Manual of Mental Disorders, Fourth Edition; GFR, Glomerular filtration rate; FPG, Fasting plasma glucose; HbA1C, Glycated hemoglobin; HDL, High-density lipoprotein; hsCRP, High-sensitivity C-reactive protein; OGTT, Oral glucose tolerance test; WC, Waist circumference.

## Competing interest

The authors declare that they have no competing interests.

## Authors’ contributions

J-HH has substantial contributions to conception and design of study, implementation of study, analysis and interpretation of data. Y-FL has substantial contributions to review the design and edit the manuscript. F-CC has substantial contributions to interpret original data and initiate the first draft of manuscript. JN-YL has substantial contributions to review and give comments on this manuscript. L-CT has substantial contributions to the conception and the original design of study, conducting the clinical study, and furnishing the final manuscript. All authors have read and approved the final manuscript.
